# Predictive value of serum testosterone for type 2 diabetes risk assessment in men

**DOI:** 10.1186/s12902-016-0109-7

**Published:** 2016-05-27

**Authors:** Evan Atlantis, Paul Fahey, Sean Martin, Peter O’Loughlin, Anne W. Taylor, Robert J. Adams, Zumin Shi, Gary Wittert

**Affiliations:** School of Nursing and Midwifery, Western Sydney University, Sydney, NSW Australia; School of Medicine, University of Adelaide, Adelaide, South Australia Australia; School of Science and Health, Western Sydney University, Sydney, NSW Australia; Freemasons Foundation Centre for Men’s Health, University of Adelaide, Adelaide, South Australia Australia; Chemical Pathology, SA Pathology, Adelaide, South Australia Australia; Population Research and Outcome Studies, University of Adelaide, Adelaide, South Australia Australia

**Keywords:** Testosterone, Diabetes, Incidence, Screening, Prognosis, ROC, Cohort

## Abstract

**Background:**

Effective prevention of type 2 diabetes (T2D) requires early identification of high-risk individuals who might benefit from intervention. We sought to determine whether low serum testosterone, a novel risk factor for T2D in men, adds clinically meaningful information beyond current T2D risk models.

**Methods:**

The Men Androgen Inflammation Lifestyle Environment and Stress (MAILES) study population consists of 2563 community-dwelling men aged 35–80 years in Adelaide, Australia. Of the MAILES participants, 2038 (80.0 %) provided information at baseline (2002–2006) and follow-up (2007–2010). After excluding participants with diabetes (*n* = 317), underweight (*n* = 5), and unknown BMI status (*n* = 11) at baseline; and unknown diabetes status (*n* = 50) at follow-up; 1655 participants were followed for 5 years. T2D at baseline and follow-up was defined by self-reported diabetes, or fasting plasma glucose (FPG) ≥7.0 mmol/L (126.1 mg/dL), or glycated haemoglobin (HbA1c) ≥6.5 %, or diabetes medications. Risk models were tested using logistic regression models. Sensitivity, specificity, positive predictive values (PPV) were used to identify the optimal cut-off point for low serum testosterone for incident T2D and the area under the receiver operating characteristic (AROC) curve was used to summarise the predictive power of the model. 15.5 % of men had at least one missing predictor variable; addressed through multiple imputation.

**Results:**

The incidence rate of T2D was 8.9 % (147/1655) over a median follow-up of 4.95 years (interquartile range: 4.35-5.00). Serum testosterone level predicted incident T2D (relative risk 0.96 [95 % CI: 0.92,1.00], *P* = 0.032) independent of current risk models including the AUSDRISK, but did not improve corresponding AROC statistics. A cut-off point of <16 nmol/L for low serum testosterone, which classified about 43 % of men, returned equal sensitivity (61.3 % [95 % CI: 52.6,69.4]) and specificity (58.3 % [95 % CI: 55.6,60.9) for predicting T2D risk, with a PPV of 12.9 % (95 % CI: 10.4,15.8).

**Conclusions:**

Low serum testosterone predicts an increased risk of developing T2D in men over 5 years independent of current T2D risk models applicable for use in routine clinical practice. Screening for low serum testosterone in addition to risk factors from current T2D risk assessment models or tools, including the AUSDRISK, would identify a large subgroup of distinct men who might benefit from targeted preventive interventions.

## Key messages

- Serum testosterone predicts risk of T2D in men independent of current risk assessment models or tools
- A cut-off point of <16 nmol/L for low serum testosterone was optimal for predicting T2D risk in men
- Over 40 % of men had low serum testosterone (<16 nmol/L)
- Additional screening for low serum testosterone would identify a large group of distinct men who might benefit from targeted preventive interventions

## Background

Type 2 diabetes (T2D) accounts for at least 90 % of all cases of diabetes and is an increasingly prevalent and debilitating disease [1]. Diabetes is currently ranked the 14th leading cause of global disease burden, and has moved up several places since 1990 [2]. The International Diabetes Federation estimates that 387 million people worldwide had diabetes in 2014, and by 2035 this figure will rise to 592 million [1]. Preventing the rising prevalence of T2D in high-income countries like Australia, where healthcare expenditure for diabetes is among the highest in the world [1], could yield significant health and economic benefits [3].

It is generally accepted that people with diagnosed T2D have progressed from ‘pre-diabetes’; an intermediate stage of impaired glucose regulation defined by impaired fasting glucose (IFG) and/or impaired glucose tolerance (IGT) [4]. The prevalence of pre-diabetes could be as high as 20 to 30 % in high-income countries [5, 6]. Progression from pre-diabetes to T2D is likely explained by non-modifiable risk factors including older age, male gender, ethnicity, and urbanisation [1]; as well as modifiable risk factors including smoking, obesity, unhealthy diet and physical activity behaviours [6–9]. Effective lifestyle programs targeting modifiable risk factors in people with pre-diabetes may delay or prevent the onset of T2D. Indeed, large-scale trials from Finland [10], China [11], and the United States [12] showed that lifestyle intervention can effectively halve the risk of developing T2D in people with pre-diabetes over three to six years.

Effective prevention of T2D requires early identification of high-risk individuals who might benefit from intervention. Screening for T2D risk factors can be cost-effective, especially when followed by lifestyle intervention [13]. There are at least seven diabetes risk models or scoring systems (often called ‘risk assessment tools’) with potential adaptation for use in routine clinical practice [14]. However, these tools may need to be updated for novel biomarkers that have emerged since these risk models were published.

Evidence from observational studies suggests that low endogenous testosterone level may be a reversible risk factor for T2D in men. For instance, a recent systematic review with meta-analysis showed that men with testosterone levels >15.5 nmol/L have a 42 % reduced risk of developing T2D compared with men with testosterone levels ≤15.5 nmol/L [15]. Cross-sectional studies in Australia show that a high proportion of men with T2D have low testosterone level, and that low testosterone level is inversely associated with glycaemia and insulin resistance [16, 17]. On average, men with T2D and metabolic syndrome (MetS) have a testosterone level that is 2.6 nmol/L lower than controls [15, 18]. Sex hormones may explain why men are more likely to develop T2D than women, as shown in several T2D risk models [19–21]. Therefore, the aim of this study was to determine whether low serum testosterone level adds clinically meaningful information beyond current T2D risk models in men, to inform guidelines and clinical practice.

## Methods

### Study design and setting

The Men Androgen Inflammation Lifestyle Environment and Stress (MAILES) prospective cohort study was established in 2009 to investigate the associations of sex steroids, inflammation, environmental and psychosocial factors with cardio-metabolic disease risk in men [22]. The MAILES study population consists of 2563 community-dwelling men aged 35–80 years at enrolment in Adelaide, Australia, from the harmonisation of two cohort studies: all participants of the Florey Adelaide Male Ageing Study (FAMAS) and a sub-set of male participants of the North West Adelaide Health Study (NWAHS). Written informed consent for participation in the study was obtained. The methods used in both FAMAS [23, 24] and NWAHS [25, 26] including the validity of subject selection for achieving a representative population sample, have been described previously.

The FAMAS is a cohort study of 1195 randomly selected men aged 35 years and over from the metropolitan region of Adelaide. Participants in the study were required to be male, aged between 35 and 80 years at the time of recruitment, living in the defined catchment area of North and West Adelaide with a connected telephone and number listed in the Electronic White Pages, be willing and able to comply with the protocol and give written, informed consent. Exclusion criteria were limited to living outside the catchment area and telephone numbers that belonged to non-residential properties. Recruiters were also instructed to exclude responders if they were: (i) of insufficient mental or physical ability to understand the requirements of participation or adequately participate; (ii) ill or otherwise incapacitated to attend clinics; (iii) currently residing in an institution (e.g. aged care facility) and (iv) non-English speaking. Prior to the study commencing, approval for the research was obtained from the Royal Adelaide Hospital Research Ethics Committee and, where appropriate, the Aboriginal Health Research Ethics Committee. The FAMAS achieved an overall response rate of 45.1 % at baseline (2002–05), and the sample was representative of the male population for most key demographics [24].

The NWAHS is a cohort study of 4060 randomly selected adults aged 18 years and over from the north-west region of Adelaide. The sample was a randomly selected population from the northern and western suburbs of Adelaide. All households in the northern and western areas of Adelaide with a telephone connected and a telephone number listed in the Electronic White Pages were eligible for selection in the study. Telephone numbers that belonged to businesses, institutions and residential care facilities were excluded from the sample. However, people who had their own telephone number and who were living in individual units attached to a nursing home were eligible to participate. Within each household, the person who had their birthday last and was aged 18 years and over, was selected for interview and invited to attend the clinic for a biomedical examination. The study excluded those people from a non-English speaking background who could not communicate sufficiently well with the telephone interviewer and who could not answer questions at the initial recruitment stage, although every effort was made to encourage family members to assist in translating. The sub-set of men aged 35 years and over was 1368. Prior to the study commencing, approval for the research was obtained from the North West Adelaide Health Service Ethics of Human Research Committee and, where appropriate, the Aboriginal Health Research Ethics Committee. The NWAHS achieved an overall response rate of 49.6 % at baseline (2000–03), and the sample was representative of the population in terms of current smoking status, body mass index (BMI), physical activity, overall health status and proportions with current high blood pressure and cholesterol readings [27].

### Study population

The series of baseline data collections that form MAILES Stage 1 include FAMAS from 2002–2005, and NWAHS from 2004–2006. The series of follow-up data collections that form MAILES Stage 2 include FAMAS from 2007–2010, and NWAHS from 2008–2010. The MAILES Stage 1 (baseline) sample was representative of the population in the northern and western regions of Adelaide [22]. A flow of the study population at each stage of the MAILES Study, with the numbers of participants drawn from the respective stages of FAMAS and NWAHS has been published [22]. Of the MAILES participants, 2038 (80.0 %) provided information at both stages of the study. Reasons for loss to follow-up (non-respondents) include death (*n* = 99), too ill to participate (*n* = 39), withdrew from the study (*n* = 141), were unable to be tracked due to changes in contact details (*n* = 77), and refused to take part in MAILES Stage 2 due to work-related and personal reasons (*n* = 169). Non-respondents in MAILES Stage 2 were more likely than respondents to report diabetes, cardiovascular disease, or depression, or to either not know or to not disclose their chronic-disease status. For self-reported assessments, non-respondents were more likely than respondents to report that they had poorer health and were current smokers or to not to provide information about these factors. For measured assessments, non-respondents were less likely than respondents to be overweight or obese, or to have central adiposity. Finally, there were no statistically significant differences between respondents and non-responders in means for serum testosterone, fasting plasma glucose (FPG), triglycerides, and high density lipoprotein cholesterol (HDL-C).

After excluding participants with diabetes at baseline (*n* = 317), underweight at baseline (*n* = 5), unknown BMI at baseline (*n* = 11), and unknown diabetes status at follow-up (*n* = 50), the final cohort sample studied consisted of 1655 participants.

### Primary outcome

T2D at baseline and follow-up was defined by self-reported medically diagnosed diabetes, or FPG ≥7.0 mmol/L (126.1 mg/dL), or glycated haemoglobin (HbA1c) ≥6.5 % according to the American Diabetes Association criteria [28], or prescriptions for medications used in diabetes based on the Anatomical Therapeutic Chemical classification system codes beginning with letters ‘A10’. Medication use was determined by linkage to the Pharmaceutical Benefits Scheme which includes details and information on medicines subsidised by the Australian Government. Plasma samples were obtained from all subjects (fasting, at clinic visits) and stored at −80 °C. Fasting plasma glucose was measured using an automated chemistry analyser system (Olympus AU5400; Olympus Corp, Tokyo, Japan). Glycated haemoglobin was measured by high-performance liquid chromatography using a spherical cation exchange gel (inter-assay coefficient of variation [CV], 2 % at 6 % of total haemoglobin).

### Predictor variables

We selected predictor variables from risk models that could be used in routine clinical practice including the Australian type 2 diabetes risk assessment tool (AUSDRISK) [19], Atherosclerosis Risk in Communities (ARIC) [29], Cambridge risk score [21], FINDRISC [30], Framingham Offspring Study [31], San Antonio Heart Study [20], and QDScore [32].

Demographic information included age, country of birth, and annual gross household income. Ethnicity was classified into two groups using country of birth data (‘Asia, Middle East, North Africa, Southern Europe’ or ‘Other’ [including Australia]). Income was classified into eight ordinal groups. Additional data collected by self-report included family history of diabetes, currently taking high blood pressure medication, doctor diagnosed cardiovascular disease (NWAHS heart attack, stroke, angina, or transient ischaemic attack; FAMAS angina, or other heart conditions), current smoking status, and leisure-time physical activity. Leisure-time physical activity data were derived from self-reported duration and intensity of physical exercise (including walking) for recreation, sport or health/fitness during the past two weeks, at the time of the interview. Total time spent in leisure-time physical activity was multiplied by intensity weights (3.3 for walking, 4.0 for moderate and 8.0 for vigorous intensity exercise) to compute metabolic equivalent-min per week (MET-mins/wk). A MET-mins/wk score of ≥540 is equivalent to 135 min or more per week of at least moderate intensity physical activity. Body weight, height and waist circumference (mean of three measures) were taken using standard anthropometric assessments. BMI was computed as weight in kilograms divided by height in metres squared. Standard international cut-off points were used to define healthy weight (BMI 18.50–24.99 kg/m^2^), overweight (BMI 25.00–29.99 kg/m^2^), and obese (BMI ≥30.00 kg/m^2^) categories. Waist circumference risk categories were classified using ethnic specific cut-off points for Asian (low ≤90 cm, medium 90–100 cm, high >100 cm), and Other (low ≤102 cm, medium 102–110 cm, and high >110 cm) based on the AUSDRISK criteria [19]. Pre-diabetes was defined by IFG using FPG levels 5.6–6.9 mmol/L (100.9–124.3 mg/dL,), or HbA1c levels 5.7–6.4 % according to American Diabetes Association criteria [28]. High blood pressure (≥140/90 mmHg) was determined by the mean of two or three readings after 10 min of seated rest. Serum lipid concentrations were determined enzymatically using a Hitachi 911 chemistry analyser (Boehringer, Germany); (inter-assay CV was 6.7 % for HDL-C, and 3.0 % for triglycerides). Cut-off points were used to classify low HDL-C (<1.0 mmol/L, or 38.6 mg/dL) and high triglycerides (>1.7 mmol/L, or 150.4 mg/dL) based on criteria proposed by the National Cholesterol Education Program, Adult Treatment Panel III [33]. Serum total testosterone was measured by an API-5000 triple-quadrupole mass spectrometer (Applied Biosystems/MDS SCIEX, Toronto, Ontario, Canada). The inter-assay coefficients of variation (CV) for serum total testosterone were 10.1 % at 0.43 nmol/L (12.4 ng/dL), 11.1 % at 1.66 nmol/L (47.8 ng/dL), and 4 % at 8.17 nmol/L (235.4 ng/dL) [34].

### Statistical analyses

Predictor variables from previously validated risk models were considered. Missing data on predictor variables were replaced through multiple imputations with five versions of the data set imputed using all predictor variables. Results for each predictor were individually tabulated for men who developed T2D and men who did not and Chi-square tests were used to confirm statistical significance. Multivariable modelling was conducted using logistic regression with developed T2D versus did not as the dichotomous outcome. The goodness of fit of the models was summarised using the Hosmer-Lemeshow (HL) Chi-square statistic: with a statistically significant (*p* < 0.05) HL statistic indicating poor fit. Akaike and Bayesian information criteria (AIC and BIC) are presented as descriptive indicators of the relative quality of competing statistical models: with smaller AICs and BICs indicating preferred models. The Nagelkerke R^2^ was monitored for sudden movement towards 1 which could indicate overfitting. The overall predictive power of the models was summarised as area under the receiver operating curve (AROC) and associated 95 % confidence intervals. The statistical significance of the difference between AROCs was tested using the method by DeLong et al. (1988) [35]. Competing cut-off points for defining low testosterone levels were visually compared on sensitivity, specificity, positive predictive power and age adjusted odds ratios from logistic regressions. Finally starting with all predictors in the model, a backwards stepwise selection algorithm (likelihood ratio P-value to exclude *P* > 0.25 and to re-enter *P* < 0.20) was applied to identify a minimal group of important independent predictors. AROCs were compared using MedCalc software (http://www.medcalc.org/); all other analyses were conducted using SPSS.

## Results

The incidence rate of T2D was 8.9 % (147/1655) over a median follow-up of 4.95 years (IQR 4.35-5.00). Table 1 shows baseline characteristics of participants in the MAILES Stage 1 cohort by T2D status at follow-up, including crude incidence and corresponding age-adjusted odds ratios. The relative incidence of T2D was significantly highest for older age and (after age-adjustment), lowest income, family history of diabetes, pre-diabetes, IFG, currently taking blood pressure medication, high blood pressure, high triglycerides, low HDL-C, obese, and high-risk waist circumference groups.

**Table 1:** Baseline characteristics of participants in the MAILES cohort by T2D status at 5 years follow-up, crude incidence and corresponding age-adjusted odds ratios

	Raw data			With multiple imputation of missing data	
	Incident T2D (*n* = 147)	No T2D (*n* = 1508)	*P*	% with T2D	Age-adjusted odds ratio (95 % CI)
Age (years)					
35–44	20	377		5.0	1
45–54	34	449		7.0	1.43 (0.80,2.52) ^a^
55–64	47	374		11.2	2.37 (1.38,4.08) ^a^
≥65	46	308	<0.001	13.0	2.82 (1.63,4.86) ^a^
Missing	0 (0.0 %)	0 (0.0 %)			
Ethnicity/Country of birth					
Asia, Middle East, North Africa, Southern Europe	15	119		11.1	0.83 (0.47,1.47)
Other (North West Europe, Americas, Oceania/Australia)	132	1387	0.329	8.7	1
Missing	0 (0.0 %)	2 (0.1 %)			
Income (AUD)					
Up to $12,000	15	72		17.1	2.89 (1.30,6.38)
$12,001 – $20,000	23	148		13.7	2.13 (1.03,4.39)
$20,001 – $30,000	24	193		11.5	1.82 (0.91,3.64)
$30,001 – $40,000	16	181		8.8	1.52 (0.74,3.13)
$40,001 – $50,000	13	188		6.5	1.16 (0.54,2.47)
$50,001 – $60,000	15	181		7.6	1.41 (0.68,2.92)
$60,001 – $80,000	18	225		8.0	1.54 (0.77,3.11)
More than $80,000	16	294	0.003	5.2	1
Missing	7 (4.8 %)	26 (1.7 %)			
Family history of diabetes					
Yes	55	410		11.9	1.80 (1.25,2.58)
No	91	1095	0.007	7.7	1
Missing	1 (0.6 %)	3 (0.2 %)			
FPG (mean, mmol/L)					
Mean (sd)	5.32 (0.71)	4.80 (0.60)	<0.001^b^		
Missing	0 (0.0 %)	10 (0.7 %)			
HbA1c (mean, %)					
Mean (sd)	5.87 (0.34)	5.49 (0.36)	<0.001^b^		
Missing	0 (0.0 %)	8 (0.5 %)			
Pre-diabetes (FPG 5.6–6.9 mmol/L or HbA1c 5.7–6.4 %)					
Yes	126	562		18.2	9.23 (5.72,14.90)
No	21	936	<0.001	2.2	1
Missing	0 (0.0 %)	10 (0.7 %)			
Impaired fasting glucose (FPG 5.6–6.9 mmol/L)					
Yes	59	142		28.7	5.63 (3.87,8.19)
No	88	1356	<0.001	6.1	1
Missing	0 (0.0 %)	10 (0.7 %)			
Currently taking blood pressure medication					
Yes	57	294		16.2	2.11 (1.43,3.11)
No	90	1209	<0.001	6.9	1
Missing	0 (0.0 %)	5 (0.3 %)			
High blood pressure (≥140/90 mmHg)					
Yes	88	629		12.2	1.77 (1.24,2.53)
No	59	874	<0.001	6.3	1
Missing	0 (0.0 %)	5 (0.3 %)			
Triglycerides (mean, mmol/L)					
Mean (sd)	1.99 (1.36)	1.70 (1.32)	0.013^b^		
Missing	0 (0.0 %)	7 (0.5 %)			
High triglycerides (>1.7 mmol/L)					
Yes	68	505		11.8	1.79 (1.27,2.54)
No	79	996	0.002	7.3	1
Missing	0 (0.0 %)	7 (0.5 %)			
HDL-C (mean, mmol/L)					
Mean (sd)	1.20 (0.31)	1.27 (0.30)	0.011^b^		
Missing	0 (0.0 %)	7 (0.5 %)			
Low HDL-C (<1.0 mmol/L)					
Yes	30	193		13.4	1.84 (1.19,2.84)
No	117	1308	0.011	8.2	1
Missing	0 (0.0 %)	7 (0.5 %)			
Diagnosed cardiovascular disease					
Yes	18	104		14.7	1.41 (0.81,2.44)
No	129	1402	0.018	8.4	1
Missing	0 (0.0 %)	2 (0.1 %)			
Total testosterone (mean, nmol/L)					
Mean (sd)	15.0 (5.9)	17.7 (5.9)	<0.001 ^b^		
Missing	10 (6.8 %)	146 (9.7 %)			
Currently smoking					
Yes	17	248		6.4	0.78 (0.46,1.32)
No	128	1257	0.136	9.4	1
Missing	2 (1.4 %)	3 (0.2 %)			
Physically inactivity (<540 METs)					
<540 METs	77	781		9.2	1.14 (0.81,1.61)
≥540 METs	62	665	0.754	8.5	1
missing	8 (5.4 %)	62 (4.1 %)			
Body mass index (kg/m2)					
Healthy weight 18.50–24.99	19	341		5.3	1
Overweight 25.00–29.99	69	768		8.2	1.57 (0.93,2.65)
Obese ≥30.00	59	399	<0.001	12.9	2.65 (1.54,456)
Missing	0 (0.0 %)	0 (0.0 %)			
Waist circumference category					
Low risk	65	925		6.5	1
Medium risk	39	361		9.7	1.42 (0.93,2.15)
High risk	42	215	<0.001	16.6	2.65 (1.74,4.02)
Missing	1 (0.7 %)	7 (0.5 %)			

Notes: *FPG* fasting plasma glucose, *IFG* impaired fasting glucose, *HbA1c* glycated haemoglobin %, *HDL-C* high density lipoprotein cholesterol, *METs* metabolic equivalents of leisure-time physical activity in the past week; Waist circumference risk categories were classified using ethnic specific cut-off points for Asian and Aboriginal (low ≤90 cm, medium 90–100 cm, high >100 cm), and Other (low ≤102 cm, medium 102–110 cm, and high >110 cm); ^a^ not age-adjusted; ^b^ independent samples *t*-test

Table 2 shows the added predictive value of low serum total testosterone compared to current T2D risk models in men over 5 years. Model 1 shows that variables from the AUSDRISK [19] resulted in good performance for predicting incident T2D. Model 2 shows additional variables from other current T2D risk models improved the AROC statistic of Model 1 (net change in AROC was 0.051 [95 % CI: 0.013,0.089], *P* = 0.009). Model 3 shows no evidence (no or very small changes in AROC and HL *χ*^2^ statistics) of improvement to Model 2 after fitting serum testosterone as a continuous variable. However, it remained an independent predictor of incident T2D (OR 0.96 [95 % CI: 0.92,1.00], *P* = 0.032) with the Nagelkerke R^2^ of 0.25.

**Table 2:** Performance of risk models for predicting 5 year risk of T2D in men

	Incidence (n/N)	AROC (95 % CI)	HL Chi-SQ statistic	*P* for HL	AIC	BIC
Risk prediction models: variables						
Model 1: Variables from AUSDRISK ^a^	147/1655	0.76 (0.72,0.80)	5.29	0.726	895	960
Model 2: Model 1 with variables from other risk models ^b^	147/1655	0.82 (0.79,0.86)	4.84	0.775	847	987
Model 3: Model 2 with total testosterone (continuous variable)	147/1655	0.82 (0.79,0.86)	4.45	0.815	844	990
Model 4: Model 2 with total testosterone (<16 vs ≥16 nmol/L)	147/1655	0.83 (0.79,0.86)	3.97	0.860	846	992
Model 5: Backwards selection model^c^	147/1655	0.82 (0.78,0.85)	5.43	0.711	825	885
Sensitivity analyses						
Model 6: Model 4 without imputation (15.5 % missing)	126/1399	0.82 (0.78,0.86)	NA	NA	NA	NA
Model 7: Model 4 for NWAHS cohort	62/820	0.79 (0.74,0.85)	NA	NA	NA	NA
Model 8: Model 4 for FAMAS cohort	85/835	0.84 (0.79,0.88)	NA	NA	NA	NA

Notes: *AROC* area under receiver operating characteristic curve (imputation with median AROC reported), *HL* Hosmer-Lemeshow, *AIC* Akaike information criterion; Bayesian information criterion, *NA* not applicable because we are not fitting a new model - just testing the existing model; ^a^ Age, ethnicity/country of birth, family history of diabetes, IFG instead of self reported high blood glucose, currently taking blood pressure medications, current smoking status, physical inactivity, waist circumference category; ^b^ Pre-diabetes, BMI category, diagnosed cardiovascular disease, high blood pressure, high triglycerides, low HDL-C, income category; ^c^ Backwards selection model started with all available variables included in Model 4, dropped if *p* > 0.25, re-entered if *p* < 0.20 (retained variables were family history of diabetes, currently taking blood pressure medications, current smoking, waist circumference category, pre-diabetes, high blood pressure, *low HDL-C* low serum testosterone <16 nmol/L); Net changes in AROC were: Model 5 vs Model 2, 0.0093 (95 % CI: −0.0032,0.0218, *P* = 0.1455)

Table 3 shows that a cut-off point of <16 nmol/L for low serum testosterone, which classified about 43 % of men, returned equal sensitivity (61.3 % [95 % CI: 52.6,69.4]) and specificity (58.3 % [95 % CI: 55.6,60.9) for predicting T2D risk, with a PPV of 12.9 % (95 % CI: 10.4,15.8). Model 4 shows there was little evidence of improvement to Model 2 after fitting low serum testosterone (<16 vs. ≥16 nmol/L) as a categorical variable (OR 1.38 [95 % CI: 0.93,2.07,], *P* = 0.114). Model 5 shows similar performance compared to Model 2 for predicting T2D risk for variables retained using backwards selection; including family history of diabetes, blood pressure medication, smoking status, waist circumference from the AUSDRISK; and high blood pressure, low HDL-C, pre-diabetes, high triglycerides and low serum testosterone (4/5 data sets). Finally, sensitivity analyses show similar AROC statistics for Model 4 without imputation (Model 6) and for Models 7 and 8 in the NWAHS and FAMAS cohorts separately.

**Table 3:** Sensitivity, specificity and positive predictive values for serum total testosterone cut-off points for predicting 5 year risk of T2D in men

	Raw data	Raw data	Raw data
Serum total testosterone cut-off points	Sensitivity, % (95 % CI)	Specificity, % (95 % CI)	Positive predictive value, % (95 % CI)
<10 vs ≥10	16.1 (10.5,23.5)	93.4 (91.9,94.6)	19.6 (13.0,28.4)
<11 vs ≥11	24.1 (17.4,32.3)	89.0 (87.2,90.6)	18.0 (12.9,24.5)
<12 vs ≥12	28.5 (21.3,36.9)	83.8 (81.8,85.7)	15.1 (11.0,20.1)
<13 vs ≥13	37.2 (29.2,45.9)	77.9 (75.6,80.1)	14.4 (11.1,18.7)
<14 vs ≥14	44.5 (36.1,53.2)	71.4 (68.9,73.8)	13.6 (10.6,17.1)
<15 vs ≥15	54.7 (46.0,63.2)	65.6 (63.0,68.1)	13.8 (11.0,17.0)
<16 vs ≥16	61.3 (52.6,69.4)	58.3 (55.6,60.9)	12.9 (10.4,15.8)
<17 vs ≥17	68.6 (60.0,76.1)	51.2 (48.6,53.9)	12.4 (10.2,15.0)
<18 vs ≥18	75.9 (67.7,82.6)	43.5 (40.9,46.2)	11.9 (9.9,14.3)
<19 vs ≥19	80.3 (72.4,86.4)	37.2 (34.7,39.9)	11.4 (9.5,13.6)
<20 vs ≥20	82.5 (74.9,88.2)	31.3 (28.8,33.8)	10.8 (9.0,12.8)

## Discussion

The results of this study confirmed that serum testosterone predicts 5 year risk of developing T2D in men (Model 3), independent of all risk factors from T2D risk assessment models or tools applicable for use in routine clinical practice, including the AUSDRISK [19–21, 29–32]. We found that an age-adjusted serum testosterone of <16 nmol/L, which was highly prevalent in the MAILES cohort (43 %), was optimal for equalising sensitivity and specificity in predicting incident T2D and has a 12.9 % PPV, which is comparable to the AUSDRISK (12.7 %) [19] and FINDRISC (13 %) [30] for optimal risk score cut-off points. This cut-off point for low serum testosterone (<16 nmol/L) is higher than that reported in a previous systematic review of prospective cohort studies on T2D risk in men (7.4–15.5 nmol/L]) [15], and also higher than that reported for predicting T2D prevalence in men (<11 nmol/L) based on the FAMAS [17].

While including serum testosterone does not improve the performance of current risk models, it remained a predictor of developing T2D after correction for all of the other predictors (Model 3). This suggests that screening for low serum testosterone would identify a large group of men otherwise not apparent with current T2D risk assessment tools, which might be clinically important for treatment decision making and resulting prognosis. Research on mechanisms suggest that low serum testosterone decreases insulin resistance indirectly by promoting metabolically favourable changes in body composition [36]; and directly by enhancing catecholamine-induced lipolysis in vitro [37] and reducing lipoprotein lipase activity and triglyceride uptake in human abdominal adipose tissue in vivo [38]. Moreover, endogenous testosterone levels correlate positively with mitochondrial indices of increased insulin sensitivity in human skeletal muscle [39], and has been shown to directly regulate pathways responsible for skeletal muscle glucose metabolism [40].

Evidence from short-term randomized controlled trials (RCTs) suggests that testosterone supplementation therapy may improve glucose control in men with, or at-risk of, low testosterone level. For instance, we meta-analysed the results of relevant studies and found that testosterone therapy improved FPG in 14 RCTs in 777 participants (standardised mean difference was −0.2 [95 % CI: −0.4,-0.1]) [41–54]; and insulin resistance in nine RCTs in 589 participants (standardised mean difference was −0.3 [95 % CI: −0.5,-0.1] for homeostasis assessment model of insulin resistance [42–47, 49, 53, 54] over short and medium terms. A recent and more relevant systematic review of RCTs (placebo-controlled) found that testosterone therapy improved insulin resistance in men with T2D and/or the metabolic syndrome (standardised mean difference was −0.34 [95 % CI: −0.51,-0.16]), over short terms [55]. In addition, evidence suggests the benefits of testosterone therapy for glucose control may be greatest when combined with lifestyle intervention [46]. This is an important therapeutic finding because there is international consensus supporting the effectiveness of lifestyle intervention in the prevention and management of T2D [56].

Conversely, testosterone therapy has been associated with serious adverse events in men. A systematic review of 27 RCTs found that testosterone therapy vs. placebo increased the risk of a cardiovascular-related event in mainly older men (pooled odds ratio was 1.54 [95 % CI: 1.09, 2.18]) [57]. However, a more recent systematic review of RCTs in mostly older men found there was an increased cardiovascular risk associated with oral testosterone therapy (pooled relative risk was 2.20 [95 % CI: 1.45,3.55]), but not with intramuscular (pooled relative risk was 0.66 [95 % CI: 0.28,1.56) or transdermal (gel or patch) testosterone therapy (pooled relative risk was 1.27 [95 % CI: 0.62,2.62]) [58]. Further research is needed to establish the safety of specific types of testosterone therapies in specific populations.

Currently, we are undertaking a Phase IIIb multicentre randomized controlled trial (double-blinded and placebo-controlled) to determine whether testosterone therapy (1000 mg testosterone undeconate) combined with lifestyle intervention will reduce the rate of T2D in men with both low testosterone and pre-diabetes or newly diagnosed T2D more than lifestyle intervention alone over two years (http://www.t4dm.org.au/). Testosterone undecanoate is registered for use in Australia for the treatment of male hypogonadism (Australian Registration Number AUST R 106946). If testosterone undecanoate is shown to be safe and effective pharmacotherapy for preventing T2D in men, then screening for low serum testosterone additional to current T2D risk assessment models (like the AUSDRISK in Australia) would identify a large subgroup of distinct men who might benefit from both targeted pharmacotherapy and lifestyle preventive interventions.

However, screening for low serum testosterone in community-based patients should be applied only to men suggestive of clinical presentations, otherwise additional blood testing would potentially cause a blowout in healthcare costs since serum testosterone level of <16 nmol/L is highly prevalent in men aged 35 years and over [59]. Furthermore, treatment decisions following confirmed screening positives will need to consider not only the optimal cut-off point for low testosterone, but also on the cost-effectiveness of adjunctive testosterone therapy, which is currently being investigated (http://www.t4dm.org.au/), as well as treatment availability.

Important quality items of this study include the large regionally representative sample of Australian men, precision of clinical measures, and the sufficient description of dropouts and non-respondents [22]. Study limitations include the reliance on a small number of self-report measures, respondent compliance, residual confounding, and misclassification of diseases and other factors potentially resulting in bias. While there were only 147 incident cases, the ratio of 100 observations per predictor variable, the relative stability of the AIC and BIC and the fact that the Nagelkerke R^2^ is much lower than 1 provide no evidence of over-fitting. Further evidence from prospective cohort studies is needed to confirm the generalizability of these findings and the applicability of screening for low serum testosterone in other male populations and specific healthcare settings.

## Conclusion

In conclusion, low serum testosterone predicts an increased risk of developing T2D in men over 5 years independent of current T2D risk models applicable for use in routine clinical practice. Screening for low serum testosterone in addition to risk factors from current T2D risk assessment models or tools, including the AUSDRISK, would identify a large subgroup of distinct men who might benefit from targeted preventive interventions.

## Abbreviations

AIC, akaike information criteria; ARIC, atherosclerosis risk in communities; AROC, area under the receiver operating characteristic; AUSDRISK, Australian type 2 diabetes risk assessment tool; BIC, bayesian information criteria; BMI, body mass index; CV, coefficient of variation; FAMAS, florey Adelaide male ageing study cohort; FINDRISC, finnish risk model; FPG, fasting plasma glucose; HbA1c, glycated haemoglobin; HDL, high density lipoprotein; HL, hosmer-lemeshow; IFG, impaired fasting glucose; IGT, impaired glucose tolerance; MAILES, Men androgen inflammation lifestyle environment and stress cohort; MET, metabolic equivalent; NWAHS, North West Adelaide health study cohort; PPV, positive predictive values; RCT, randomised controlled trial; T2D, type 2 diabetes.

## References

[1] International Diabetes Federation. IDF Diabetes Atlas. 6th Edn. © 2013 International Diabetes Federation. 2013.

[2] Murray CJ (2012). Disability-adjusted life years (DALYs) for 291 diseases and injuries in 21 regions, 1990–2010: a systematic analysis for the Global Burden of Disease Study 2010. Lancet.

[3] AIHW (2008). Diabetes: Australian facts 2008. Diabetes series no. 8. Cat. no. CVD 40; 150 pp.

[4] Genuth S (2003). Follow-up report on the diagnosis of diabetes mellitus. Diabetes Care.

[5] Cowie CC (2009). Full accounting of diabetes and pre-diabetes in the U.S. population in 1988–1994 and 2005–2006. Diabetes Care.

[6] Magliano DJ (2008). Glucose indices, health behaviors, and incidence of diabetes in Australia: the Australian Diabetes, Obesity and Lifestyle Study. Diabetes Care.

[7] Danaei G (2011). National, regional, and global trends in fasting plasma glucose and diabetes prevalence since 1980: systematic analysis of health examination surveys and epidemiological studies with 370 country-years and 2.7 million participants. Lancet.

[8] Atlantis E (2009). Chronic disease trends due to excess body weight in Australia. Obes Rev.

[9] Alberti KG (2007). International Diabetes Federation: a consensus on Type 2 diabetes prevention. Diabet Med.

[10] Tuomilehto J (2001). Prevention of type 2 diabetes mellitus by changes in lifestyle among subjects with impaired glucose tolerance. N Engl J Med.

[11] Pan XR (1997). Effects of diet and exercise in preventing NIDDM in people with impaired glucose tolerance. The Da Qing IGT and Diabetes Study. Diabetes Care.

[12] Knowler WC (2002). Reduction in the incidence of type 2 diabetes with lifestyle intervention or metformin. N Engl J Med.

[13] Gillies CL (2008). Different strategies for screening and prevention of type 2 diabetes in adults: cost effectiveness analysis. BMJ.

[14] Noble D (2011). Risk models and scores for type 2 diabetes: systematic review. BMJ.

[15] Ding EL (2006). Sex differences of endogenous sex hormones and risk of type 2 diabetes: a systematic review and meta-analysis. JAMA.

[16] Grossmann M (2008). Low testosterone levels are common and associated with insulin resistance in men with diabetes. J Clin Endocrinol Metab.

[17] Atlantis E (2011). Testosterone and modifiable risk factors associated with diabetes in men. Maturitas.

[18] Brand JS (2011). Testosterone, sex hormone-binding globulin and the metabolic syndrome: a systematic review and meta-analysis of observational studies. Int J Epidemiol.

[19] Chen L (2010). AUSDRISK: an Australian Type 2 Diabetes Risk Assessment Tool based on demographic, lifestyle and simple anthropometric measures. Med J Aust.

[20] Stern MP (2002). Identification of persons at high risk for type 2 diabetes mellitus: Do we need the oral glucose tolerance test?. Ann Intern Med.

[21] Rahman M (2008). A simple risk score identifies individuals at high risk of developing Type 2 diabetes: a prospective cohort study. Fam Pract.

[22] Grant JF (2014). Cohort profile: The men androgen inflammation lifestyle environment and stress (MAILES) study. Int J Epidemiol.

[23] Martin S (2007). Cohort profile: the Florey Adelaide Male Ageing Study (FAMAS). Int J Epidemiol.

[24] Martin SA (2007). The Florey Adelaide Male Ageing Study (FAMAS): design, procedures & participants. BMC Public Health.

[25] Grant JF (2009). Cohort Profile: The North West Adelaide Health Study (NWAHS). Int J Epidemiol.

[26] Grant JF (2006). The North West Adelaide Health Study: detailed methods and baseline segmentation of a cohort for selected chronic diseases. Epidemiol Perspect Innov.

[27] Taylor AW (2006). Do people with risky behaviours participate in biomedical cohort studies?. BMC Public Health.

[28] Diagnosis and classification of diabetes mellitus. Diabetes Care. 2010;33 Suppl 1:S62-9. doi:10.2337/dc10-S062. 10.2337/dc10-S062 PMC2797383 20042775

[29] Schmidt MI (2005). Identifying individuals at high risk for diabetes - The Atherosclerosis Risk in Communities study. Diabetes Care.

[30] Lindstrom J (2003). The diabetes risk score: a practical tool to predict type 2 diabetes risk. Diabetes Care.

[31] Wilson PWF (2007). Prediction of incident diabetes mellitus in middle-aged adults - The Framingham Offspring Study. Arch Intern Med.

[32] Hippisley-Cox J (2009). Predicting risk of type 2 diabetes in England and Wales: prospective derivation and validation of QDScore. BMJ.

[33] Grundy SM (2005). Diagnosis and Management of the Metabolic Syndrome: An American Heart Association/National Heart, Lung, and Blood Institute Scientific Statement: Executive Summary. Circulation.

[34] Harwood DT (2009). Development and validation of a sensitive liquid chromatography-tandem mass spectrometry assay to simultaneously measure androgens and estrogens in serum without derivatization. Clin Chim Acta.

[35] DeLong ER (1988). Comparing the areas under two or more correlated receiver operating characteristic curves: a nonparametric approach. Biometrics.

[36] Isidori AM (2005). Effects of testosterone on body composition, bone metabolism and serum lipid profile in middle-aged men: a meta-analysis. Clin Endocrinol.

[37] Xu XF (1991). Testosterone increases lipolysis and the number of beta-adrenoceptors in male rat adipocytes. Endocrinol.

[38] Marin P (1995). Assimilation and mobilization of triglycerides in subcutaneous abdominal and femoral adipose tissue in vivo in men: effects of androgens. J Clin Endocrinol Metab.

[39] Pitteloud N (2005). Relationship between testosterone levels, insulin sensitivity, and mitochondrial function in men. Diabetes Care.

[40] Haren MT (2011). Testosterone modulates gene expression pathways regulating nutrient accumulation, glucose metabolism and protein turnover in mouse skeletal muscle. Int J Androl.

[41] Boyanov MA (2003). Testosterone supplementation in men with type 2 diabetes, visceral obesity and partial androgen deficiency. Aging Male.

[42] Caminiti G (2009). Effect of Long-Acting Testosterone Treatment on Functional Exercise Capacity, Skeletal Muscle Performance, Insulin Resistance, and Baroreflex Sensitivity in Elderly Patients With Chronic Heart Failure: A Double-Blind, Placebo-Controlled, Randomized Study. J Am Coll Cardiol.

[43] Cornoldi A et al. Effects of chronic testosterone administration on myocardial ischemia, lipid metabolism and insulin resistance in elderly male diabetic patients with coronary artery disease. Int J Cardiol. In Press, Corrected Proof. 10.1016/j.ijcard.2008.12.107 19361872

[44] Emmelot-Vonk MH (2008). Effect of testosterone supplementation on functional mobility, cognition, and other parameters in older men: a randomized controlled trial. JAMA.

[45] Gopal RA et al. Treatment of hypogonadism with testosterone in type 2 diabetes mellitus. Endocrine Practice 2010;Rapid Electronic Articles in Press 1–20. 10.4158/EP09355.OR 20150021

[46] Heufelder AE (2009). Fifty-two-Week Treatment With Diet and Exercise Plus Transdermal Testosterone Reverses the Metabolic Syndrome and Improves Glycemic Control in Men With Newly Diagnosed Type 2 Diabetes and Subnormal Plasma Testosterone. J Androl.

[47] Kapoor D (2006). Testosterone replacement therapy improves insulin resistance, glycaemic control, visceral adiposity and hypercholesterolaemia in hypogonadal men with type 2 diabetes. Eur J Endocrinol.

[48] Malkin CJ (2006). Testosterone therapy in men with moderate severity heart failure: a double-blind randomized placebo controlled trial. Eur Heart J.

[49] Malkin CJ (2007). The effect of testosterone on insulin sensitivity in men with heart failure. Eur J Heart Fail.

[50] Marin P (1992). The effects of testosterone treatment on body composition and metabolism in middle-aged obese men. Int J Obes Relat Metab Disord.

[51] Marin P (1993). Androgen treatment of abdominally obese men. Obes Res.

[52] Nair KS (2006). DHEA in elderly women and DHEA or testosterone in elderly men. N Engl J Med.

[53] Schroeder ET (2004). Effects of androgen therapy on adipose tissue and metabolism in older men. J Clin Endocrinol Metab.

[54] Svartberg J (2008). Testosterone treatment in elderly men with subnormal testosterone levels improves body composition and BMD in the hip. Int J Impot Res.

[55] Grossmann M (2014). Effects of testosterone treatment on glucose metabolism and symptoms in men with type 2 diabetes and the metabolic syndrome: a systematic review and meta-analysis of randomized controlled clinical trials. Clin Endocrinol.

[56] International Diabetes Federation et al. Global Guideline for Type 2 Diabetes. © International Diabetes Federation, 2012.

[57] Xu L (2013). Testosterone therapy and cardiovascular events among men: a systematic review and meta-analysis of placebo-controlled randomized trials. BMC Med.

[58] Borst SE (2014). Cardiovascular risks and elevation of serum DHT vary by route of testosterone administration: a systematic review and meta-analysis. BMC Med.

[59] Atlantis E (2009). Demographic, physical and lifestyle factors associated with androgen status: the Florey Adelaide Male Ageing Study (FAMAS). Clin Endocrinol.

